# Math difficulties in attention deficit hyperactivity disorder do not originate from the visual number sense

**DOI:** 10.3389/fnhum.2022.949391

**Published:** 2022-10-28

**Authors:** Giovanni Anobile, Mariaelisa Bartoli, Gabriele Masi, Annalisa Tacchi, Francesca Tinelli

**Affiliations:** ^1^Department of Neuroscience, Psychology, Pharmacology and Child Health, University of Florence, Florence, Italy; ^2^Department of Developmental Neuroscience, IRCCS Fondazione Stella Maris, Pisa, Italy

**Keywords:** ADHD, numerosity, approximate number system, mathematical abilities, numerical cognition

## Abstract

There is ample evidence from literature and clinical practice indicating mathematical difficulties in individuals with ADHD, even when there is no concomitant diagnosis of developmental dyscalculia. What factors underlie these difficulties is still an open question. Research on dyscalculia and neurotypical development suggests visual perception of numerosity (the number sense) as a building block for math learning. Participants with lower numerosity estimation thresholds (higher precision) are often those with higher math capabilities. Strangely, the role of numerosity perception in math skills in ADHD has been neglected, leaving open the question whether math difficulties in ADHD also originate from a deficitary visual number sense. In the current study we psychophysically measured numerosity thresholds and accuracy in a sample of children/adolescents with ADHD, but not concomitant dyscalculia (*N* = 20, 8–16 years). Math abilities were also measured by tasks indexing different mathematical competences. Numerosity performance and math scores were then compared to those obtained from an age-matched control group (*N* = 20). Bayesian statistics indicated no difference between ADHD and controls on numerosity perception, despite many of the symbolic math tasks being impaired in participants with ADHD. Moreover, the math deficits showed by the group with ADHD remained substantial even when numerosity thresholds were statistically regressed out. Overall, these results indicate that math difficulties in ADHD are unlikely to originate from an impaired visual number sense.

## Introduction

Attention deficit hyperactivity disorder (ADHD) is characterized by pervasive and severe symptoms of inattention, hyperactivity, and impulsivity having a direct negative impact on social, academic, or occupational functioning (DSM5, American Psychiatric Association., 2013). Together with those primary deficits there is also indication of difficulties in school-based mathematical achievements (Zentall et al., 1994; Kaufmann and Nuerk, 2008; Colomer et al., 2013; Tosto et al., 2015; Kuhn et al., 2016; Ganor-Stern and Steinhorn, 2018; Orbach et al., 2020; von Wirth et al., 2021) but the processes underlying the observed mathematical difficulties are still unclear. One possibility is that mathematical difficulties originate from a deficitary numerosity perception system, which has been named “number sense” (Dehaene, 2011). The main aim of the current study is to investigate this possibility.

### Numerosity perception and symbolic mathematical abilities

Numerosity perception refers to humans’ (and other animals’) capacity to estimate numerical quantities (sets of elements) when serial counting is not allowed. Usually counting is prohibited through the rapid presentation of the visual stimuli (few milliseconds), and the classical tasks measuring this function are based on psychophysical procedures requiring the comparison of the relative numerosity of different groups of objects. Numerosity perception obeys Weber Law (Ross, 2003), a psychophysical law describing the relationship between the physical and the perceived stimulus magnitude. According to Weber Law, the discrimination threshold between two stimuli (the smallest noticeable difference) scales linearly with the stimulus intensity. One of main signature of numerosity perception is that discriminability between two ensembles depends on their numerical ratio (Ross, 2003; Anobile et al., 2016a). This ratio can be described by Weber Fraction (Wf), the main behavioral parameter targeting numerosity perception precision. This parameter can be derived by normalizing the discrimination threshold to the target (or perceived) numerosity and can be conceptualized as an index of numerical acuity, with relatively higher values indicating lower precision and likely reflecting higher sensory noise (Lasne et al., 2019). The minimal discriminable numerical ratio (Weber fraction) decreases with age, reflecting the refinement of the numerosity system (Piazza, 2010; Halberda et al., 2012). More in details, the developmental trajectory is particularly steep during the early development reaching a plateau around 20 years of age. Newborns can discriminate ratios of 1:3 (Wf ≈ 3), at 1 year of life the ratio decreases to 2:3 (Wf≈ 0.5). Adults, starting from approximately around 20 years of age, can discriminate two ensembles with a numerical ratio of 7:8 (Wf ≈ 0.15). Weber fraction has been frequently used in the literature as a developmental index of the numerosity system, providing a useful tool to describe typical and atypical trajectories (Piazza et al., 2010, 2013). Despite remaining controversial (Leibovich et al., 2017), a fascinating theory suggests that this non-symbolic perceptual function might act as an early start-up tool on which later symbolic math abilities build (Piazza, 2010). The rationale is that mathematical meaning could be mapped onto the pre-existing non-symbolic counterpart (numerosity). For atypical development of the numerosity system, mapping between digits and their non-symbolic counterpart will be impaired, impacting the development of mathematical skills. Evidence for this notion comes from correlational studies showing that children with more precise numerosity perception (lower Wf) often perform better on school-based math tasks, such as mental calculation (Halberda et al., 2008; Chen and Li, 2014; Anobile et al., 2016b; Schneider et al., 2017). Clinical studies also point to a link between numerosity perception and math abilities with dyscalculic children (a neurodevelopmental disorder impairing math learning) often showing lower numerosity precision (Piazza et al., 2010; Mazzocco et al., 2011; Anobile et al., 2018), suggesting that dyscalculia might originate from a deficitary numerosity system (Butterworth et al., 2011). Whether a numerosity perception deficit also underlies mathematical difficulties in ADHD is still unexplored.

### Mathematical abilities and attention deficit hyperactivity disorder

Individuals with ADHD, even without comorbid dyscalculia, often report math difficulties. A relatively recent review systematized the data from 34 studies measuring math abilities in children and adults with ADHD (Tosto et al., 2015). Out of the selected studies, 30 (88%) reported significant negative association between ADHD symptoms and mathematical scores. Most of these studies (76%) showed that the negative association remains significant even after controlling for IQ, age, socioeconomic status, and other factors, indicating a specific deficit.

Mathematics is not a monolithic construct, but composed of many different skills, including mental calculation, counting, fact retrieval, transcoding and many others. Although there is consensus in showing difficulties in learning mathematics in ADHD, the results are rather mixed in identifying which mathematics sub-components are impaired. To understand math difficulties in ADHD deeper, Colomer et al. (2013) investigated several numerical abilities in primary school children with ADHD but not comorbid dyscalculia. The results revealed that many children have severe difficulties (–2 SD below age mean) in counting (∼30%) and mental calculation (∼25%). There were also a high percentage of children showing moderate difficulties (–1 < SD < –2 below age mean) in facts retrieval (∼30%) and written calculation (∼20%). Overall, the ADHD sample showed more severe math difficulties than what was expected in the general population. Interestingly children with ADHD showed an unimpaired performance on a number comparison task (which digit was numerically larger) requiring magnitude encoding. Results indicating a spared numerical magnitude process were also showed by Kuhn et al. (2016) and von Wirth et al. (2021). More in details while both studies showed a spared ability to map numbers onto space according to their magnitude (number line task), von Wirth et al. (2021) confirmed an unimpaired ability to compare and order digits according to their relative numerical magnitude. At odds with these results (Kaufmann and Nuerk, 2008) observed a significant difference between ADHD and neurotypicals in a task asking children to discriminate between the magnitude of two Arabic one-digit numerals appearing simultaneously on a computer screen (which is bigger?), with children with ADHD committing more errors and having longer response times, indicating a deficit in numerical magnitude processing. Regarding other mathematical subcomponents, the results also do not agree. For example, while Colomer et al. (2013) found severe difficulties in counting and mental calculation, Kaufmann and Nuerk (2008) observed no significant impairments on similar tasks. The same results’ discrepancies can be observed for facts retrievals, with some studies showing moderate impairments (Colomer et al., 2013) but others not (von Wirth et al., 2021). Discordant results also exist about counting skills. While Colomer et al. (2013) and Kuhn et al. (2016) showed lower counting abilities in children with ADHD, two other studies showed no difficulties (Kaufmann and Nuerk, 2008; von Wirth et al., 2021). On transcoding abilities, the literature seems more coherent, generally showing none or moderate deficits (Kaufmann and Nuerk, 2008; Colomer et al., 2013; Kuhn et al., 2016; von Wirth et al., 2021). In sum, the existing literature point to major mathematical difficulties of ADHD in counting and calculation, however, the results are mixed.

### The current study

Overall, these (and many other) investigations (as well as the clinical practice) clearly indicate math difficulties in ADHD. However, the results are heterogeneous, the source of those difficulties is still unclear, and the potential role played by the numerosity system is completely neglected. The aim of the current study is to fill this gap. To this aim, we enrolled children/adolescents with ADHD but no concurrent dyscalculia nor any past or ongoing pharmacological treatments. Numerosity precision (thresholds: Weber fraction) and accuracy were psychophysically measured by a categorization task (see methods) and math abilities by means of an age-standardized paper-and-pencil battery designed for the diagnosis of dyscalculia. The performance on these tasks was compared to those obtained from an age-matched neurotypical sample.

Given that the literature indicates math difficulties in individuals with ADHD, we expect lower performance. As previous evidence (Colomer et al., 2013) showed relatively stronger impairments on counting, fact retrievals, and number writing abilities, we expect more severe impairments on these mathematical tasks. Since there is no previous evidence regarding numerosity perception, we do not have a specific hypothesis. However, if numerosity perception contributes to math deficits in ADHD, we expect higher discrimination thresholds in the group with ADHD, compared to controls. Moreover, the arithmetic difficulties in the group with ADHD should be substantially explained by inter-individual variability in the psychophysically measured numerosity performance. Finally, if numerosity perception and ADHD are linked, we expect a correlation between numerosity thresholds and ADHD symptomatology.

## Materials and methods

### General procedure

This dataset is part of a larger project aimed to measure attention, time and numeracy in children and adolescents with neurodevelopmental disorders. Participants were tested by experienced psychologists and child neuropsychiatrists in a clinical setting at the Stella Maris Foundation Institute in Pisa (Italy). More specifically, participants were individually tested in a quiet, dimly lit room carefully avoiding environmental distraction factors. During the psychophysical assessment, participants were comfortably sitting on a chair in front of a desk where a monitor was placed. Visual stimuli were created with Psychophysics toolbox for Matlab (Brainard, 1997; Pelli, 1997; Kleiner et al., 2007) and displayed on a 60 Hz—17” screen monitor placed at a viewing distance of 57 cm. Before the test, participants with and without a diagnosis of ADHD were informed about the study activities, with particular a focus on assuaging competitive/evaluating feelings. Numerosity perception and math abilities were usually tested on the same day.

### Participants

As the main question of the study was to test whether ADHD (as dyscalculia) is characterized by a numerosity perception deficit, and since there are not available studies on numerosity perception in ADHD, we calculated the required sample size extracting the effect size (*d* = 1.2) from a recent study employing similar methodologies with a sample of individuals with and without dyscalculia and an age range similar to the one considered here (Anobile et al., 2018). A power analysis to detect a difference between the averages derived from two independent samples of equal size (two-tailed *t*-test), an alpha of 0.05 and a required power of 0.95 provides an estimate of 20 individuals needed for each group. For this study we enrolled and test forty children/adolescents: 20 with a diagnosis of ADHD (6 female, 14 males, mean age = 11.2 year-old, age range 8–16) and 20 with neurotypical development (11 female, 9 males, mean = 11.2 year-old, range 8.1–16.2). Inclusion criteria for the group with ADHD were: clinical diagnosis of ADHD based on DSM-5, a total intelligence quotient (TIQ) evaluated with the Wechsler Intelligence Scale for Children-IV (Wechsler, 2003) above 75, no neurological or sensory deficits, no psychiatric comorbidities, no current or past pharmacological treatment. ADHD symptoms were measured with the Conners Parent Rating Scale (CPRS, Conners et al., 1998). According to Conners et al. (1998) CPRS reliability coefficients alphas for the seven scales in the age range considered here (8–16 yrs) ranges from 0.75 to 0.93. General clinical symptoms were measured with the Clinical Global Impression-Severity scale (CGI-S, William, 1976) and the Children Global Assessment Scale (CGAS, Shaffer et al., 1983). According to Shaffer et al. (1983) CGAS inter-rater reliability was 0.84 while six months test-retest reliability 0.85. Three participants with ADHD met the criteria for a diagnosis of developmental dyslexia (not excluded from the analyses). Absence of autism was clinically screened with the Child behavior Checklist 6-18 (for a similar procedure see: Duarte et al., 2003; Biederman et al., 2010; Ooi et al., 2011). The clinical assessment for dyscalculia was based on DSM-5 and supplemented by the Italian guidelines (Cornoldi et al., 2012) according to which, in order to meet diagnostic criteria for a diagnosis of developmental dyscalculia, children must show a performance below the 5th percentile (or –2 SD) on at least 50% of the tasks on a specific and comprehensive battery for the assessment of mathematical abilities (such as the one used here); the problems with math should be persistent, resistant to treatments, and limiting the child’s daily life. The participants of the current study (both with and without a diagnosis of ADHD) did not meet any of these criteria. ADHD non-verbal reasoning skills were computed by a combined index of WISC-IV measuring Visual Perceptual Reasoning (IRP). The IQ of four ADHD participants was measured by an external independent institute and, for those participants, we were unable to calculate IRP. The control group consisted of a sample of neurotypical children/adolescents matched for chronological age. The inclusion criteria for this group were: no medical history, negative neuro-psychiatric examination, and no learning difficulties (reported by parents). Non-verbal reasoning abilities were evaluated by Raven Colored Progressive Matrix-CPM or Progressive Matrix-PM, depending on chronological age (Belacchi et al., 2008). Detailed individual raw scores for each scale are available online at doi: 10.5281/zenodo.6411431.

### Mathematical abilities

Mathematical abilities were measured with six representative tasks extracted from an Italian battery for the diagnosis of dyscalculia (BDE-2, Biancardi et al., 2016). The tasks were individually performed by each participant, requiring ∼30 min on average, and providing information on counting, transcoding, mental calculation, and digit magnitude processing. For each test, and separately for ADHD and controls, we measured *z*-scores relative to the normative age-standardized data provided by the test (for a similar procedure see Colomer et al., 2013; Anobile et al., 2018).

The tests were: (1) *Counting*. Participants were asked to count aloud between 80 and 140 or between 1 and 40 (depending on age). Participants were then asked to count again but from the largest to the smallest number until he/she reached the time taken in the ascending counting task. The score was the total numbers stated correctly. (2) *Numbers reading*. Participants were asked to read aloud Arabic numbers (48 or 18 depending on age). Numbers were arranged in different lists each composed of 12 integer numbers (three, four, five, or six digits). The score was the total numbers correctly read within 60 s. (3) *Numbers writing*. Participants were asked to transcribe numbers read out by the experimenter (three to six digits). The score was given by the number of digits correctly written. (4) *Mental multiplications*. Participants were asked to provide the result (within 3 secs) of simple multiplications (e.g., 2 × 3) read by the examiner. Eighteen multiplications were administered to each participant and the score was the total correct answers provided. (5) *Mental additions and subtractions*. Participants were asked to provide the result (within 30 secs) of 9 addition and 9 subtractions (e.g., 27 + 14, 43–12) read by the examiner. The score was the total correct answers provided. 6) *Choose the largest*. Participants were given a sheet with 18 sets of three Arabic numbers (one to five digits) and asked, for each set, to mark the largest number. The score was a combination of both speed and accuracy.

### Numerosity perception

Numerosity perception was psychophysically measured with a categorization task (for a similar procedure see Anobile et al., 2022). On each test trial, while participants fixated on a central point on the screen, a dot ensemble was briefly presented (500 ms, to prevent counting). Participants were asked to verbally categorize the stimulus as containing “many” or “few” dots. The response was recorded by the experimenter by an appropriate keypress. To provide an idea of the numerical range, before the testing phase, four initial “anchoring” trials were provided. On these trials the lower (N8) and higher (N32) numerosities were presented twice each and the participants were told that those ensembles corresponded to the range extremes (no responses were required). The dots (0.25° of diameter, 50% white and 50% black to balance luminance) were randomly scattered (avoiding dots overlap) within a virtual circular area (12° diameter) around the central fixation point. The geometric mean of the tested range was 16 dots with stimuli ranging from one octave above and below, divided into 11 steps: 8, 9, 11, 12, 14, 16, 18, 21, 24, 28, and 32. Each stimulus was presented 4 times, in a single session, for a total of 44 trials. On each trial, one stimulus was presented (randomly chosen by the range) and children were asked to categorize it. The proportion of “many” responses was plotted against the stimuli magnitude (in log scale) and fitted with a cumulative Gaussian error function. The 50% point of the fit provided an estimate of the point of subjective equality (PSE), the difference in numerosity between the 50 and 75% points provided the just notable difference (JND), which was used to estimate Weber Fractions (10^JND-1).

### Data analyses

Data were analysed by Bayesian statistics (*t*-tests, ANCOVAs and Pearson correlations), measuring Bayes Factors, the ratio of the likelihood of the alternative to the null hypothesis (see Supplementary Appendix for data analyses with different prior width), and reporting them as base ten logarithms (LBF) (Jeffreys, 1961; Lavine and Schervish, 1999; Jarosz and Wiley, 2014). For ANCOVA we report LBF_*inclusion*_ indicating how much the data are likely to occur from a model including that specific factor, compared to models not including them. By convention (Jarosz and Wiley, 2014), LBF > 0.5 is considered substantial evidence in favor of the alternative hypothesis (difference between groups in this case) and LBF < −0.5 substantial evidence for the null hypothesis (no difference). Absolute values greater than 1 are considered strong evidence, and greater than 2 definitive. Statistical assumptions were checked before reporting the results. When comparing the groups on the “chose the largest” and the “number writing” tasks, we detected a violation of normality in both groups. For these comparisons we used a Bayesian non-parametric test (Mann–Whitney *t*-test). A violation of normality was also detected in the correlational analysis between numerosity Wf and the aggregate math score (for both groups) as well as in the correlational analysis between numerical performance and ADHD symptomatology (Table 1). For these analyses, we report Bayesian Kendall’s non-parametric correlation. Male female ratio difference was tested with a Bayesian Multinomial Test. Visual Perceptual Reasoning (IRP) and Raven matrices scores were both converted into *z*-scores (according to the normative age-standardized data provided by the tests manuals). This procedure was performed to make the two measures comparable between groups. Because of time constraints, one neurotypical participant completed 4 out of six math tasks, two neurotypical participants completed 5 out of six math tasks and one participant with ADHD did not complete any of the mathematical tasks (see Table 2). Missing data were left empty and cases excluded per dependent variable (see Supplementary Appendix for data analyses with imputation). Data were analysed by JASP (Version 0.16.1) and Matlab software. Matlab was used to fit the numerosity task data with psychometric functions to estimate thresholds and PSEs. JASP was used for all the other statistical tests.

**TABLE 1:** Descriptive statistics on numerosity perception.

	Groups	*N*	Mean (SD)	Min	Max	Kurtosis	Skewness
Weber fraction	ADHD	20	0.177 (0.09)	0.02	0.43	1.32	1.01
	Controls	20	0.143 (0.07)	0.03	0.35	1.6	0.995
PSE	ADHD	20	15.63 (2.38)	11.76	19.48	–0.99	–0.06
	Controls	20	14.76 (1.97)	11.5	18.23	–0.86	0.04

N, sample size; Mean, between participants average; SD, standard deviation; Min, minimum; Max, maximum; Weber fraction, numerosity perceptual threshold; PSE, point of subjective equality from the numerosity perception task.

**TABLE 2:** Descriptive statistics on math abilities (*z*-scores).

Math tasks	Groups	*N*	Mean (SD)	Min	Max	Kurtosis	Skewness
Counting	ADHD	18	−0.33(1.18)	–2.1	2.1	–0.66	0.34
	Controls	20	0.78 (0.84)	–0.99	2	–0.41	–0.61
Reading	ADHD	18	−0.85(1.23)	–3.68	1.11	0.37	–0.61
	Controls	20	−0.13(1.28)	–3.44	1.39	0.46	–0.87
Writing	ADHD	18	−0.28(0.98)	–2.66	0.93	1.08	–1.14
	Controls	20	0.82 (0.90)	–2.23	2.06	6.48	–2.07
Mental multiplications	ADHD	19	−0.94(1.07)	–3.7	0.96	1.33	–0.70
	Controls	19	0.12 (0.76)	–1.59	1.43	–0.07	–0.49
Mental addition/subtraction	ADHD	19	−0.19(1.06)	–1.96	1.66	–1.03	–0.02
	Controls	19	0.42 (1.17)	–1.89	1.84	–0.45	–0.71
Choose the largest	ADHD	18	−0.58(1.62)	–4.2	0.98	–0.06	–1.07
	Controls	18	0.34 (0.47)	–0.71	1.05	–0.18	–0.57
Math aggregate index	ADHD	19	−0.49(0.71)	–1.94	0.73	–0.50	–0.34
	Controls	20	0.41 (0.58)	–0.93	1.28	–0.19	–0.38

N, sample size; Mean, between participants average z-scores; SD, standard deviation; Min, minimum; Max, maximum, counting, reading, writing, mental multiplications, mental addition/subtraction, choose the largest, symbolic mathematical tasks (see section “Materials and methods” for details), math aggregate index, average z-score combining the performance on all symbolic mathematical tasks.

## Results

### Demographical variables and attention deficit hyperactivity disorder cognitive profile

The ADHD and control groups did not differ in age (LBF = −0.51) and male/female ratio (LBF = −0.47). Regarding non-verbal reasoning scores we found no sufficient evidence for a difference or no difference between groups (Visual Perceptual Reasoning, IRP; ADHD: mean = –0.15, SD = 0.9; Raven test Controls: mean = 0.42, SD = 0.95; LBF = 0).

**FIGURE 1:**
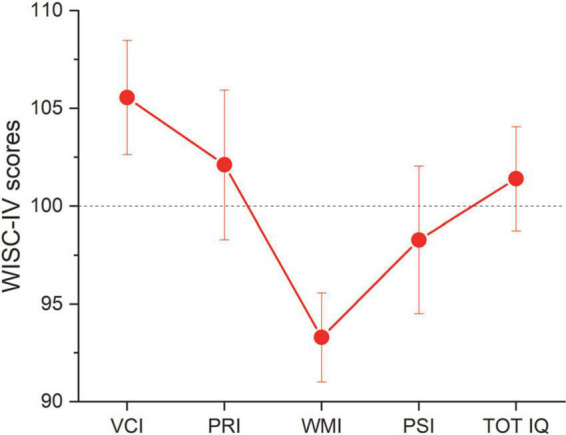
WISC-IV: Wechsler Intelligence Scale for Children, 4th edition. CVI, Verbal Comprehension index; PRI, perceptual reasoning index; WMI, working memory index; PSI, processing speed index; TOT IQ, full scale quotient. Dashed line report normative data. Error bars report ± 1 s.e.m.

Figure 1 reports the average cognitive profile (WISC-IV scores) for the group with ADHD. In line with previous studies (Mayes and Calhoun, 2006; Ünal et al., 2021), the cognitive profile was not homogeneous with the working memory (WMI) and processing speed indexes (PSI) showing stronger deviation from the normative data (dashed line), compared to the other indexes (see Table 3 for descriptive statistics).

**TABLE 3:** WISC-IV for the ADHD group: Wechsler Intelligence Scale for Children, 4th edition.

CVI	Mean = 105.56; SD = 12.43
PRI	Mean = 102.11; SD = 16.22
WMI	Mean = 93.3; SD = 9.4
PSI	Mean = 98.3; SD = 16
TOT IQ	Mean = 101.4; SD = 12

CVI, verbal comprehension index; PRI, perceptual reasoning index; WMI, working memory index; PSI, processing speed index; TOT IQ, full scale quotient; Mean, between subject’s average; SD, standard deviation.

### Mathematical abilities

**FIGURE 2:**
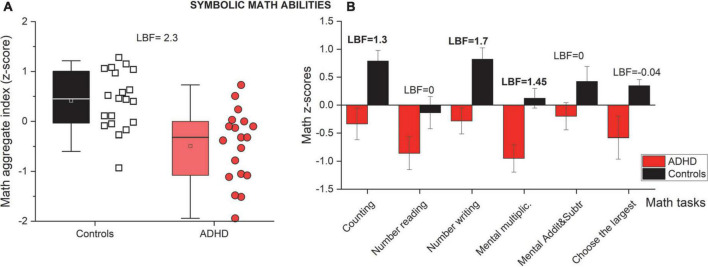
Mathematical abilities. **(A)** Whisker plots reporting mathematical abilities as an aggregate index (average *z*-score across six math tests). Color and Whisker conventions as before. **(B)** Participants’ average math scores divided into the six different math tests for controls *(black)* and ADHD groups *(red)*. Error bars report ± 1 s.e.m. Strong evidence (LBF > 1) in favor of the alternative hypothesis (difference between groups) are indicated in bold.

Figure 2A reports whisker plots of participants’ math abilities as an aggregate index obtained by averaging the *z*-scores obtained in the six math tests (see Table 2 for descriptive statistics). From inspection, it is evident that participants with ADHD performed poorly compared to controls. The participants’ average *z*-scores (aggregate index) for controls was 0.4 (SD = 0.6) while for participants with ADHD it was –0.5 (SD = 0.7). Statistical analyses (*t*-test) confirmed the difference (LBF = 2.3) suggesting definitive evidence for a group difference. Given the heterogeneity of the math tests used, we also looked at each task separately. Figure 2B reports participants’ average *z*-scores for each of the six math tests, divided by group (controls and ADHD). Overall, participants with ADHD performed worse compared to controls, across all the tasks (red bars always lower compared to black bars), but some of the tasks were particularly impaired (Table 2). More decisive evidence for an impairment (LBF > 1) was seen in tasks involving counting, number writing and mental multiplications. The other three tasks (number reading, mental addition/subtractions and choose the larger) were more similar across the groups, providing no statistical evidence for a true difference (LBF < 0.5). These results were robust if imputations were used (see Supplementary Appendix).

### Numerosity perception

**FIGURE 3:**
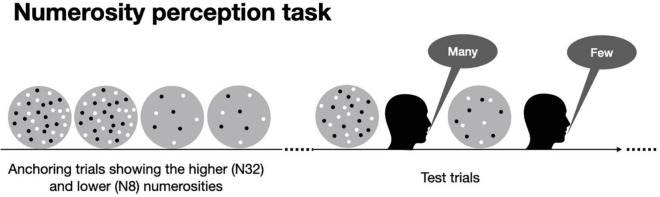
Numerosity perception task. A block consisted of four initial “anchoring” trials in which the more numerous (N32) and the less numerous (N8) stimuli were presented twice (participants were told that those numerosities corresponded to the range extremes). After that the testing phase started. On each test trial, while participants fixated on a central point on the screen a dot ensemble (with a numerosity randomly drawn from a pre-defined distribution: N8-32, geometric mean N16) was briefly presented (500 ms) and participants were asked to verbally categorize the stimulus as containing “many” or “few” dots.

**FIGURE 4:**
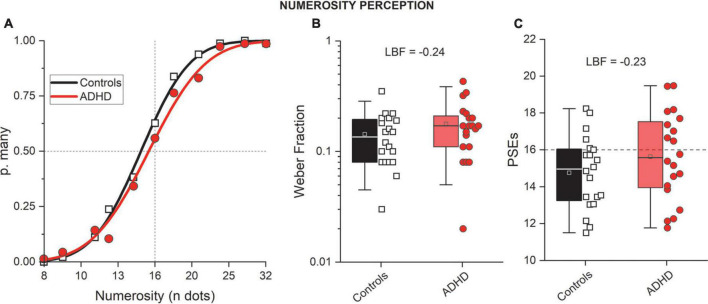
Numerosity perception. **(A)** Psychometric functions (aggregate data) reporting the probability of “many” (p. many) responses as a function of the test numerosity divided for controls (black, squares) and participants with ADHD (red, circles). **(B,C)** Whisker plots reporting numerosity thresholds (Weber fractions, **B**) and accuracy (PSEs, **C**). Color conventions as before. Whisker parameters. Box: 25–75 percentiles; Whisker: 5–95 percentiles; Line: average; Square: median.

The results on mathematical abilities showed an impairment in children/adolescents with ADHD. We than looked at the non-symbolic counterpart: numerosity. Numerosity perception was measured with a visual categorization task (Figure 3). All participants were able to perform the psychophysical numerosity task, producing ordered psychometric functions. Figure 4A shows psychometric functions obtained by aggregating all the data together across participants. It is evident that the curves for participants with (red) and without (black) a diagnosis of ADHD have similar slopes, indicating a similar precision level (thresholds, Weber Fractions). The numerosity corresponding to where the curves cross the 50% is the Point of Subjective Equality (PSE), an index of estimations accuracy. The aggregate data (Figure 4A) again shows similar performance between groups, suggesting that both groups were able to compare the ongoing stimuli with the anchors and the stimuli average. In this case participants with ADHD were slightly more veridical compared to controls, showing a smaller shift (bias) from the range center.

**FIGURE 5:**
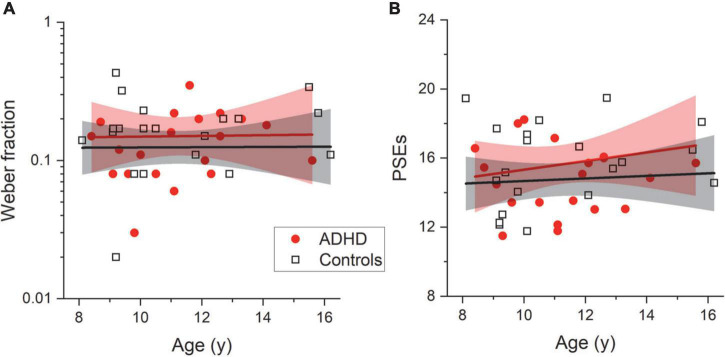
Developmental trajectories. Numerosity discrimination thresholds (Wf, **A**) and point of subjective equality (PSEs, **B**) as a function of chronological age for both controls (black squares) and participants with ADHD (red circles). Lines report best linear fitting with associated confidence bands (95%).

The fitting procedure was then applied to the data provided by each participant (see Table 1 for descriptive statistics). Figures 4B,C report whisker plots for numerosity thresholds (Weber Fractions, Wf) and PSEs, separately for the two groups. Single subject data are reported by symbols. From inspection, it is evident that the two groups performed similarly on both parameters. This was confirmed by statistical analyses indicating the null hypothesis (no difference) as more likely compared to the alternative (LBF = –0.24 and LBF = –0.23 for Weber fractions and PSEs, respectively). Both Weber fraction and PSEs were not correlated with age (Wf Controls: LBF = –0.52; Wf ADHD: LBF = –0.88; PSEs Controls: LBF = –0.52; PSEs ADHD: LBF = –0.41, Figure 5). This set of results suggests similar numerosity perception abilities between participants with and without ADHD.

### Link between mathematical ability and numerosity perception

**FIGURE 6:**
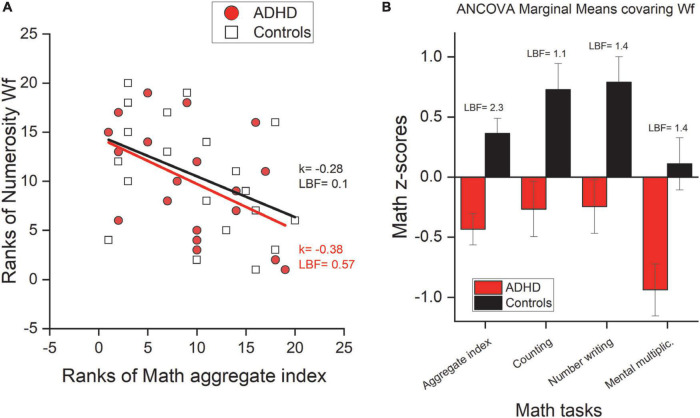
Mathematical abilities and numerosity perception. **(A)** Kendall rank correlation between numerosity precision (Wf) and math abilities (aggregate index). **(B)** ANCOVA marginal means of math *z*-scores obtained after having regressed out numerosity precision (Wf). Error bars report ± 1 s.e.m.

The results so far suggest an intact numerosity system despite deficits in mathematical skills. Although this indicates a certain degree of independence of the two functions (numerosity and mathematics), we looked at inter-individual differences within the two groups. Separately for ADHD and controls, we correlated numerosity perception precision (Wf) with formal mathematical skills. Figure 6A shows a negative trend in both groups, with participants who show more precise numerosity estimates (lower Wf) also showing higher mathematical skills (*k* = –0.28, LBF = 0.09 and *k* = –0.39, LBF = 0.57 for controls and ADHD, respectively, these results were robust if imputations were used).

To investigate this point further we asked whether the deficits shown by participants with ADHD in some of the mathematical tests were explained by inter-individual variability in numerosity estimation thresholds. To this aim we re-analysed the differences between groups in those mathematical tests where participants with ADHD showed a marked deficit and on the aggregate index, but this time entering numerosity estimation precision (Wf) as a covariate (ANCOVA). Figure 6B shows the marginal means of the ANCOVA test. Even when numerosity thresholds (Wf) were regressed out, the difference between groups in the arithmetic tasks remains substantial (all LBF > 1) and virtually identical to those obtained without entering any covariates, confirming again that the arithmetic deficits are unlikely to originate from the numerosity system (these results were robust if imputations were used, see Supplementary Appendix).

As a final check, we looked at the role of domain-general factors. To this aim, we repeated the ANCOVA analysis, but this time entering (simultaneously) numerosity thresholds (Wf), non-verbal reasoning scores (IRP, see methods), age and sex as covariates. Even when the contribution of all these factors was regressed out, the group differences on math abilities remained substantial (Aggregate Index LBF = 2.2; Counting LBF = 0.8; Number writing LBF = 1.7; Mental multiplication LBF = 1.4).

### Correlations between numerical skills and attention deficit hyperactivity disorder clinical symptoms

Within the sample with ADHD, we ran correlations between numerosity performance (thresholds and PSEs) math aggregate index, and both general (CGI-S, CGAS) and specific clinical symptoms (the average of the four CPRS indexes, see section “Materials and methods” for details). For the CGAS test, which provided range scores, we transformed the ranges into categorical values reflecting the symptoms severity (following the test manual: from 1 to 10 with one indicating no symptoms and 10 indicating very severe symptoms). The analyses revealed no meaningful correlations (see Table 4).

**TABLE 4:** Kendall’s correlations.

Measure	CGI-S	CGAS	CPRS
Weber fraction	k = 0.13 LBF = –0.4	k = 0.164 LBF = –0.33	k = –0.05 LBF = –0.48
PSE	k = 0.09 LBF = –0.46	k = –0.193 LBF = –0.25	k = 0.15 LBF = –0.36
Math aggregate index	k = –0.07 LBF = –0.5	k = 0.147 LBF = –0.37	k = 0.35 LBF = 0.198

CGI-S, Clinical Global Impression-Severity scale; CGAS, Children Global Assessment Scale; CPRS, Conners Parent Rating Scale; Weber fraction, numerosity perceptual threshold; PSE, point of subjective equality from the numerosity perception task; math aggregate index, average z-score combining the performance on all symbolic mathematical tasks; k, Kendall’s correlation coefficient; LBF, LBF, Log10 Bayes Factor; LBF > 0.5, substantial evidence for H_1_ (correlation).

## Discussion

The current study is the first to measure visual numerosity perception in individuals with a diagnosis of ADHD. Results indicate similar thresholds (Weber fraction) and accuracy levels between neurotypical and age-matched participants with ADHD. Replicating previous studies, although the sample with ADHD recruited in the current study did not included individuals with dyscalculia, we found lower scores (compared to controls) along all the math tasks, with more severe difficulties in three out of six math tasks (counting, fact retrievals and numbers writing). Interestingly, when numerosity thresholds were regressed out, these impairments in math remained substantial. Overall, the results indicate that math difficulties in ADHD are unlikely to originate from an impaired visual numerosity system (number sense).

### Numerosity perception and mathematical abilities

Numerosity is considered a primary perceptual visual feature such as motion, color, orientation, and others (Burr and Ross, 2008; Anobile et al., 2016a; Burr et al., 2017). The number of visual items in the scene is spontaneously perceived (Cicchini et al., 2016, 2019) even without an extensive school-based mathematical experience (Ferrigno et al., 2017; Anobile et al., 2019). Visual numerosity thresholds (Weber fraction) are considered to reflect the sensory noise associated with the internal representation of numerical quantities (Lasne et al., 2019), sharply decreasing in the first years of life (Halberda et al., 2012). Numerosity thresholds, together with natural maturation, also change as a function of cultural factors, such as the opportunity to benefit from an adequate arithmetic schooling (Piazza et al., 2013). Even at the same educational level, numerosity thresholds have demonstrated a specific portion of individual differences in math scores (Halberda et al., 2008), and are often impaired in individuals with developmental dyscalculia (Piazza et al., 2010; Mazzocco et al., 2011; Anobile et al., 2018). These results lead to the idea that visual numerosity might act as an early precursor for the optimal development of later school-based math abilities (Piazza, 2010). The current results suggest that children/adolescents with ADHD have a typical numerosity sensory noise level when compared to age-matched controls, suggesting a spared visual number sense. Moreover, in addition to showing unimpaired thresholds, participants with ADHD demonstrated unimpaired accuracy levels. The task used in this study was a categorization task requiring the comparison of ongoing visual stimuli with the previously memorized range of extremes as well as constructing a running average of the incoming stimulation. An incorrect processing of these features would have led to a shift in the psychometric curves (bias in accuracy). Participants with ADHD showed the same accuracy compared to controls, demonstrating an intact ability to memorize an accurate trace of numerosity, as well as an intact ability to build an accurate numerical running average. These results were by no means guaranteed. A recent study using the same psychophysical technique to investigate auditory time perception in ADHD found deficits on thresholds as well as on accuracy, compared to controls (Anobile et al., 2022). Finally, results on developmental trajectories indicate that the sparing on numerosity thresholds and accuracy was constant along the age range tested here (8–16 years), speaking against any developmental delay. Although the results indicate that for our sample with ADHD but not dyscalculia there is no impairment in the number sense, some limitations must be considered, advising caution in the interpretation and generalization of this result. One of the limitations is linked to the sample size and participants features. To draw firm conclusions for a truly spared numerosity perception in ADHD, as well as on the nature of the link between numerosity and math in ADHD, replication studies with larger and more heterogenous (e.g., ADHD with dyscalculia, different developmental stage) samples are needed.

At odds with the spared numerosity visual system, symbolic mathematical abilities were impaired in our sample of ADHD. This is largely in line with the literature. ADHD and dyscalculia are often associated and individuals with ADHD but not comorbid dyscalculia often report math difficulties (DuPaul et al., 2013). Our results, as well as confirming overall lower math performance in individuals with ADHD but no concomitant dyscalculia (see introduction for details), also perfectly replicated previous studies showing much more marked impairments on counting, fact retrievals, and number writing abilities (Colomer et al., 2013). Another interesting point emerging from the results is that, although there was no difference in numerosity discrimination performances between the two groups, within both samples we found a correlation between numerosity thresholds and overall mathematical abilities. In line with many previous studies, participants with higher mathematical skills also showed lower numerosity discrimination thresholds (higher precision) confirming a common source of variance between these two skills (Halberda et al., 2008; Piazza, 2010; Anobile et al., 2013, 2016b; Piazza et al., 2013).

Another issue emerging from the current results is the null correlation, within the group with ADHD, between the clinical symptomatology severity and mathematical scores. This null result might indicate that the level of mathematical competence does not explain inter individual variance in the severity of ADHD clinical symptomatology. It should be noted, however, that the clinical tests used are mainly aimed at gathering information about the global child’s behavior and functioning in a variety of different life contexts. The “global” nature of the assessment, while clinically fundamental, may not be sufficiently specific and detailed in the measurement of the cognitive factors underlying mathematical abilities, such as executive functions (detailed below).

### The role of domain general functions

If not numerosity, what is driving math underachievement in ADHD? The current study was specifically designed to address the role of the visual number sense and thus, with the current data, we cannot directly answer this question. However, the available literature on math abilities in ADHD suggests that domain-general functions—such as poor executive functioning (including working memory)—seems a good candidate. Executive functions refer to the processes required to monitor and control thought and action, allowing flexible responses to environment requests, and is composed of several skills such as inhibition, shifting, working memory and updating (Diamond, 2013). There is large evidence in the literature suggesting executive functions as a key process that sustains math learning (Cragg et al., 2017; Bellon et al., 2019; Gashaj et al., 2019; Spiegel et al., 2021; Agostini et al., 2022; Santana et al., 2022) as well as indicating poor executive functions in ADHD (Barkley, 1997). The results on the cognitive profile of our participants with ADHD confirmed this evidence, showing a clear weakness on the WISC-IV working memory index. In line with this, deficits on counting, fact retrievals, and number writing abilities have been previously linked to poor automaticity and executive functioning in ADHD (Colomer et al., 2013; Orbach et al., 2020). Similarly, inhibition, shifting, and updating has been found to explain a specific portion of math variance, above those explained by digit magnitude processing (Bellon et al., 2019), along the neurotypical development. Although in the current work we have not directly measured the role played by executive functions (and an exhaustive discussion of how these relate to arithmetic abilities is beyond the scope of this paper), we would speculate that they underlie the current pattern of results. Another interesting point emerges from the data: participants with ADHD performed similarly to controls on a task selecting the numerically largest digit number (among others). This task is thought to tap into the number magnitude comprehension, and has been previously found strongly linked to numerosity thresholds in both neurotypicals and dyscalculic children (Piazza et al., 2010; Anobile et al., 2013, 2016b), suggesting common resources. That both numerosity thresholds and digit magnitude comprehension are spared in our group with ADHD is in line with the idea that both symbolic and non-symbolic magnitude processing might be relatively spared, compared to other math sub-processes. Interestingly, a spared numerosity system in the face of deficitary math abilities is reminiscent of what has been shown with preterm children/adolescents. Prematurity is a well-known risk factor for math underachieving (Isaacs et al., 2001; Aarnoudse-Moens et al., 2009; Taylor et al., 2009). However, there is evidence for a spared numerosity system in both school-age (Tinelli et al., 2015) and newborn preterm individuals (Anobile et al., 2021), again suggesting that the source of math difficulties was not to be sought in the numerosity system. Similarly to what we are speculating here regarding math difficulties in ADHD, it has been previously suggested that those encountered by preterm subjects might not derive from domain-specific deficits in the visual number sense, but from poorer domain-general functions (Simms et al., 2015), that also involve early perceptual-motor abilities (De Rose et al., 2013). While the current results show mathematical impairments in individuals with attentional deficits (ADHD) but not dyscalculia, the role of attention has also been documented in children with dyscalculia who do not meet the criteria for ADHD but have severe attentional deficits. Kißler et al. (2021) described two clusters of children with dyscalculia (but not ADHD) that differed by their degree of mathematical deficit severity. Importantly, the group with more severe mathematical impairment also had more severe attentional deficits, indicating that attention could be a key factor to identify different subtypes of dyscalculia. In summary, the transition between children with ADHD and deficits in arithmetic (without a diagnosis of dyscalculia) and children with deficits in arithmetic and attention (but without a diagnosis of ADHD) could be fluent, depicting a continuum. Looking at attentional and executive skills together with both symbolic and non-symbolic (numerosity perception) mathematical abilities could thus represent an informative avenue for the development of efficient and individualized strategies to support numeracy skills. It should be noted here that since we did not measure executive functions and attentional skills, their role in the current study was indirectly inferred from the existing literature. To fully understand the pattern of results obtained here, future studies should simultaneously measure numerosity perception, math abilities and the different components of executive functions. Finally, different test procedures were used in the ADHD group (WISC-IV) compared to the control group (Raven’s Matrices) to measure non-verbal reasoning skills. While previous evidence on neurotypical samples indicates similar scores between the two tests, a clinical sample with a diagnosis of autism showed strong score discrepancies, with Raven’s Matrices providing higher scores compared to Wechsler scales (Dawson et al., 2007). Although we do not know whether this discrepancy is also generalisable to other neurodevelopmental disorders (such as ADHD), we might have underestimated the ADHD group’s non-verbal abilities.

### Neuronal correlates

Given the behavioral nature of the current work, we cannot say anything definitive about the neural structures underlying the observed pattern of results, however, these are generally in line with a previously suggested idea that the frontal cortex could be relatively more impaired compared to the parietal cortex in individuals with ADHD (Aman et al., 1998). As mentioned before, the results on math performance replicated previous studies showing relatively more severe impairments on counting, fact retrievals, and number writing abilities, compared to other tasks (Colomer et al., 2013). The authors proposed that these more pronounced deficits could reflect a higher demand on automatization skills, a process usually problematic in individuals with ADHD and tapping on frontal neural structures (Miller, 2000). Regarding the non-symbolic counterpart, numerosity perception has been repeatedly linked to the functioning of the parietal cortex (Piazza et al., 2004; Lasne et al., 2019). In particular, the intraparietal sulcus has been suggested as a key area for the encoding of cardinality, i.e., the relative numerical magnitude (Piazza and Eger, 2016). In the current study, this last process was particularly involved in the numerosity discrimination task and in the mathematical task requiring the selection of the numerically largest digit within a set (“chose the largest”). The results showed similar performance between individuals with and without ADHD across both tasks suggesting a relatively spared parietal functioning.

## Conclusion

To summarize, by confirming math difficulties in ADHD but showing a spared visual numerosity system, our results indicate a different etiology underlying the arithmetic difficulties encountered in dyscalculia and those encountered by individuals with ADHD (but not concomitant dyscalculia). These results have implications for the design of math interventions in the ADHD population, encouraging the targeting of domain-general functions.

## Data availability statement

The datasets presented in this study can be found in online repositories. The names of the repository/repositories and accession number(s) can be found in the article/Supplementary material.

## Ethics statement

The studies involving human participants were reviewed and approved by the Ethics Committee of the Meyer’s Hospital (n. 248/2020 ID-DNATN, 20 October 2020 “Attention, time and numeracy in children and adolescents with neurodevelopmental disorders”). Written informed consent to participate in this study was provided by the participants’ legal guardian/next of kin.

## Author contributions

GA, MB, and FT: conceptualization. GA, MB, GM, and FT: methodology, resources, and writing—review and editing. GA: software, writing—original draft preparation, and visualization. GA and FT: formal analysis. GA, GM, and FT supervision. MB, GM, and FT: project administration. All authors read and agreed to the published version of the manuscript.
