# Supplementary material for: Math difficulties in attention deficit hyperactivity disorder do not originate from the visual number sense
Source: Front Hum Neurosci. 2022 Oct 28;16:949391. doi: 10.3389/fnhum.2022.949391 (PMC9649814; doi:10.3389/fnhum.2022.949391)
Supplement: Supplementary file 1 [file Data_Sheet_1.docx]

**Supplementary Material**

As reported in the data analysis section, missing data were left empty and cases excluded per dependent variable. However, for those variables where we had missing data, we also performed the analyses with imputation. As an imputation method, we used the replacement of the missing data with the average of that variable, calculated on the data from the specific sample (ADHD or Controls). Appendix tables 1, 2 and 3 show the results obtained with and without imputation. For all the statistical tests the results remained unchanged.

| **Appendix Table 1**  **Bayesian independent sample t-test contrasting ADHD and Controls.** | | |
| --- | --- | --- |
| **variable** | **LBF no imputation** | **LBF with imputation** |
| Non-verbal reasoning scores | 0 | 0.23 |
| Math aggregate Index | 2.31 | 2.98 |
| Counting | 1.3 | 1.42 |
| Reading | 0 | 0.05 |
| Writing | 1.7 | 1.53 |
| Mental multiplications | 1.45 | 1.55 |
| Mental addition/subtraction | 0 | 0.01 |
| Choose the largest | −0.04 | −0.02 |
| Non-verbal reasoning scores= Controls: Z-scores obtained from Raven Colored Progressive Matrix-CPM or Progressive Matrix-PM (depending on age), ADHD: Z-scores computed on a combined index of WISC-IV measuring Visual Perceptual Reasoning, Math aggregate index= average z-score combining the performance on all symbolic mathematical tasks (Counting, Reading, Writing, Mental multiplications, Mental addition/subtraction, Chose the largest; see methods for a detailed description), LBF no imputation= Log10 Bayes Factor obtained leaving missing data empty and cases excluded per dependent variable, LBF with imputation= Log10 Bayes Factor obtained replacing the missing data with the average of that variable, calculated on the data from the specific sample (ADHD or Controls). | | |

| **Appendix Table 2**  **Correlations between Mathematical scores (aggregate index) and numerosity Weber Fraction** | | |
| --- | --- | --- |
| **group** | **LBF no imputation** | **LBF with imputation** |
| ADHD: | k= –0.39, LBF= 0.57 | k= –0.4, LBF= 0.72 |
| k= Kendall’s Correlations, LBF no imputation= Log10 Bayes Factor obtained leaving missing data empty and cases excluded per dependent variable, LBF with imputation= Log10 Bayes Factor obtained replacing the missing data with the average of that variable, calculated on the data from the specific sample (ADHD or Controls). | | |

| **Appendix Table 3**  **ANCOVA contrasting mathematical performance between ADHD and controls, controlling for Weber Fraction.** | | |
| --- | --- | --- |
| **variable** | **LBF no imputation** | **LBF with imputation** |
| Math aggregate Index | 2.3 | 2.9 |
| Counting | 1.1 | 1.3 |
| Writing | 1.4 | 1.55 |
| Mental multiplications | 1.4 | 1.5 |
| Math aggregate index= average z-score combining the performance on all symbolic mathematical tasks (Counting, Reading, Writing, Mental multiplications, Mental addition/subtraction, Chose the largest; see methods for a detailed description). Counting, Writing, Mental multiplications= mathematical tasks showing the strongest difference between the two groups (ADHD or Controls), LBF no imputation= Log10 Bayes Factor obtained leaving missing data empty and cases excluded per dependent variable, LBF with imputation= Log10 Bayes Factor obtained replacing the missing data with the average of that variable, calculated on the data from the specific sample (ADHD or Controls). | | |

As a second check, we looked at the robustness of the main results as a function of prior width. As in the main text we report the results with no imputation, this check has been performed on that dataset. When comparing the performance of ADHD and controls on mathematical and cognitive scores, numerosity perception parameters (Wf and PSEs) as well as demographical features, we used the default JASP prior scale (Cauchy, scale= 0.7). Appendix table 4 shows the results of the same analyses paired with those obtained using a wide (1) and ultra-wide (1.4) prior. For all the statistical tests the results remained similar across prior width values.

| **Appendix Table 4**  **Bayesian independent sample t-test contrasting ADHD and Controls as a function of prior width.** | | | |
| --- | --- | --- | --- |
| **Variable** | **LBF with**  **Prior= 0.7** | **LBF with**  **Prior= 1** | **LBF with**  **Prior= 1.4** |
| Age | −0.51 | −0.63 | −0.76 |
| Non-verbal reasoning scores | 0 | 0 | −0.11 |
| Math aggregate Index | 2.31 | 2.36 | 2.36 |
| Counting | 1.3 | 1.3 | 1.28 |
| Reading | 0 | −0.05 | −0.14 |
| Writing | 1.7 | 1.34 | 1.13 |
| Mental multiplications | 1.45 | 1.47 | 1.45 |
| Mental addition/subtraction | 0 | −0.08 | −0.17 |
| Choose the largest | −0.04 | −0.18 | −0.25 |
| Weber fraction | −0.24 | −0.33 | −0.44 |
| PSE | −0.23 | −0.33 | −0.45 |
| LBF= Log10 Bayes Factor, Prior= prior width used to calculate t-test, Age= chronological age, Non-verbal reasoning scores= Controls: Z-scores obtained from Raven Colored Progressive Matrix-CPM or Progressive Matrix-PM (depending on age), ADHD: Z-scores computed on a combined index of WISC-IV measuring Visual Perceptual Reasoning, Math aggregate index= average z-score combining the performance on all symbolic mathematical tasks (Counting, Reading, Writing, Mental multiplications, Mental addition/subtraction, Chose the largest; see methods for a detailed description), Weber fraction= Numerosity perceptual threshold, PSE= Point of subjective equality from the numerosity perception task. | | | |
